# Exploring behaviors, treatment beliefs, and barriers to oral chemotherapy adherence among adult leukemia patients in a rural outpatient setting

**DOI:** 10.1186/s13104-018-3935-z

**Published:** 2018-11-29

**Authors:** C. Suzanne Lea, Sulochana Bohra, Tiffanie Moore, Chelsea Passwater, Darla Liles

**Affiliations:** 1 Department of Public Health, Brody School of Medicine, East Carolina University, Mailstop 660, Greenville, NC 27834 USA; 2 Division of Hematology/Oncology, Brody School of Medicine, East Carolina University, Greenville, NC 27834 USA; 3 Vidant Cancer Services, Vidant Medical Center, Greenville, NC 27834 USA; 4 Present Address: 3900 Paramount Parkway, Morrisville, NC USA

**Keywords:** Oral chemotherapy, Medication adherence, Survey, Adult, Hematology, Rural, ASK-12

## Abstract

**Objective:**

Adherence to oral chemotherapy is essential for patients with chronic myeloid leukemia (CML) and multiple myeloma (MM) to remain in remission. Few studies have used a Likert-type scale to measure medication adherence in CML and MM patients. We applied a validated treatment adherence tool, the ASK-12 (Adherence Starts with Knowledge^®^) survey, which assessed inconvenience and forgetfulness, treatment beliefs, and medication-taking behaviors recorded on a five-point Likert-type scale at two visits.

**Results:**

A medication adherence survey was administered to 42 newly diagnosed or pre-existing CML or MM patients at two outpatient oncology clinics affiliated with an academic medical center in rural eastern North Carolina. Thirty-one patients completed surveys at visit 1 and visit 2 (median 4.5 months apart). Most patients were treated for MM (65%), were non-Hispanic black (68%) and female (58%). Within subscales, mean adherence scores decreased between visits, signaling better adherence. Overall, visit scores were correlated (0.63, *p *= 0.001). Forgetting to take medication sometimes was the most common reason for non-adherence. Medication costs were not a barrier for MM patients. Greater patient–provider informed decision-making was identified as an opportunity for quality improvement among CML patients. The ASK-12 survey provided a strategy to obtain robust information on medication adherence.

**Electronic supplementary material:**

The online version of this article (10.1186/s13104-018-3935-z) contains supplementary material, which is available to authorized users.

## Introduction

Multiple myeloma (MM) mortality rates are almost twice as common in blacks as compared to whites [1], while chronic myelogenous leukemia (CML) mortality rates are more similar [2]. The emergence of oral chemotherapies allows MM and CML to be managed as chronic diseases [3, 4], thereby reducing survival disparities [5, 6]. However, patients must adhere to complex oral medication regimens to remain in remission [7–9]. Medication adherence, which is defined as the extent to which a patient acts in accordance with the prescribed interval and dose of a regimen [10], requires the patient to consume daily or weekly oral medicines for many consecutive months to remain disease free [11]. Non-adherence has been associated with cancer resurgence, development of medication resistance, and increased consumption of healthcare resources [12]. Insufficient data exists on estimates of CML and MM medication adherence in non-white populations, particularly in community-based cancer centers [11].

Consensus is lacking for a gold standard approach to measure oral medication adherence in hematology patients [3, 11, 13]. Several reviews advocate that simultaneous measures of both objective (direct observation, patient monitoring systems) and subjective (self-reported surveys) approaches [14, 15] are beneficial to assess adherence in CML patients [16]. Studies of adherence in MM patients are rare because the oral chemotherapies are relatively new [4].

In community-based, rural hospitals, objective methods, such as monitoring prescription refills or electronic bottle cap opening technology, are often neither feasible nor practical. For our patients with low literacy, we sought a validated, short survey as an efficient method to obtain feedback about challenges to medication adherence during a patient encounter. This paper presents our findings using the 12-question “Adherence Starts with Knowledge Survey^®^” (ASK-12) [17] to assess medication adherence. We aimed to explore adherence behaviors, treatment beliefs, and barriers to adherence among patients with MM and CML.

## Main text

### Methods

**Fig. 1:**
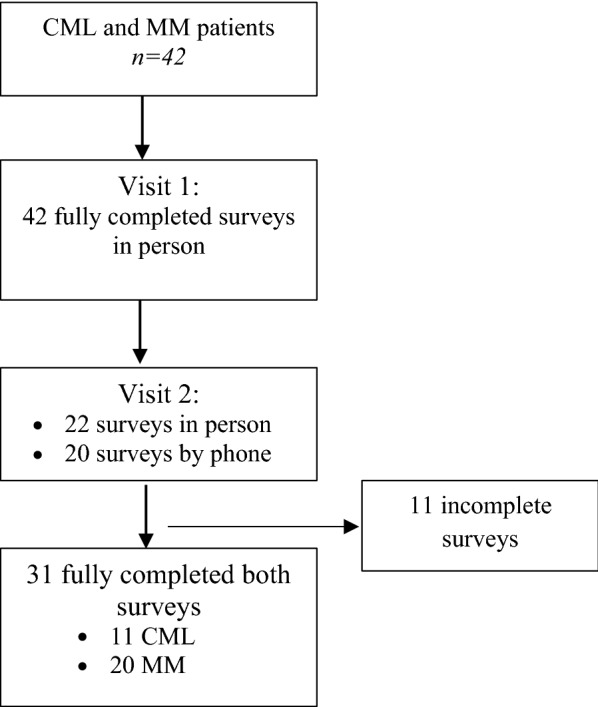
Participant flow chart completing ASK-12 survey

This exploratory study assessed barriers to medication adherence using the ASK-12 survey, conducted at two time points in the same patients receiving oncology care at two outpatient clinics affiliated with an academic medical center in rural, eastern North Carolina. A non-probability, convenience sample of adult hematology–oncology patients scheduled for routine follow-up care during a specified time interval served as the basis for the sample size. When a patient arrived for a scheduled visit, the usual clinical care and assessment from oncologist occurred, including an educational session with the oncology nurse. During routine outpatient visits, an oncology physician, clinical research specialist, or oncology nurse explained the quality improvement goals to adults (≥ 18 years of age) and administered the visit 1 survey in-person to 42 CML and MM patients. At the next scheduled appointment, the visit 2 survey was administered in-person for 22 patients and by phone for 20 patients unable to complete visit 2 survey in-person (Fig. 1). Patients provided verbal agreement to complete the surveys to help improve delivery of their care and education. All surveys were administered in English between March 2015 and January 2016. The study was approved by the University and Medical Center Institutional Review Board (UMCIRB 14-000847) at East Carolina University.

#### ASK-12 survey

While the Morisky Medication Adherence Scale (MMAS) has been widely used for self-reported oral medication adherence in CML patients treated at large academic centers [11], we selected the ASK-12 survey due to several design features relevant for our low-literacy patient population. The ASK-12 survey, which was derived from the ASK-20 survey [17, 18], was validated to assess barriers to adherence and problems with adherence behavior. The survey also assesses treatment beliefs and was visually designed to be easily interpreted during a brief outpatient visit. The 12-question survey, designed to be self-administered, has five-Likert-type responses (strongly agree, agree, neutral, disagree, and strongly disagree) with three subscales: (1) inconvenience and forgetfulness, (2) treatment beliefs, and (3) medication-taking behavior. The ASK-12 survey has good reliability and construct validity with other medication-taking adherence scales designed for use in patients on long term oral therapies, such as hypertension or diabetes [17, 19]. The survey demonstrated strong correlation with the dichotomous, four-item MMAS, adequate validity, and internal consistency (Cronbach’s α was 0.75) [17, 19].

#### Assessing adherence

Inconvenience and forgetfulness (subscale 1), includes questions 1–3 and relates to taking medications as prescribed. Responses with “strongly disagree” scored 1 (most adherent), and responses with “strongly agree” scored 5 (most non-adherent) with a range from 3 to 15. For inconvenience and forgetfulness, mean scores of ≤ 6 were interpreted as adherent and scores ≥ 12 were non-adherent.

Treatment beliefs (subscale 2), includes questions 4–7, ranging from “strongly disagree” (scored 5, most non-adherent) to “strongly agree” (scored 1, most adherent) with a range from 4 to 40. For treatment beliefs, mean scores ≤ 8 were interpreted as adherent and ≥ 16 as non-adherent.

Medication-taking behavior (subscale 3), was assessed by questions 8–12, ranging from “in the last week” to “never”. Responses “in the last week” scored 5 (most non-adherent), whereas “never” scored 1 (most adherent) with a range from 5 to 25.

The total score ranged from 12 to 60, which was computed by summing responses for all questions. Patients with a total score of 12 were most adherent with fewer barriers to adherence, whereas patients with a total score of 60 were least adherent.

Data analysis was limited to patients that completed surveys at visit 1 and visit 2. Descriptive statistics including means, medians, standard deviations (SD) and Pearson’s correlation coefficient were computed. Tests of differences in proportion were used to compare visit 1 to visit 2 (Table 1). All tests were two-tailed with *p *< 0.05. Analysis was conducted using Stata release 14 (College Station, TX). The analytic dataset is available from the corresponding author on reasonable request.

**Table 1:** Descriptive statistics for three ASK-12 subscales among adult leukemia patients

Subscale	Visit 1			Visit 2			R^e^	*p*-value^e^
	Mean (SD)	Median	Range	Mean (SD)	Median	Range		
All patients (n = 31)								
Inconvenience^a^/forgetfulness	6.23 (2.7)	6	3–11	5.68 (2.4)	5	3–10	0.53	0.002
Treatment beliefs^b^	7.48 (3.0)	8	4–19	7.19 (2.5)	8	4–14	0.41	0.02
Behavior^c^	7.81 (3.7)	7	5–21	7.29 (3.5)	5	5–17	0.38	0.03
Total^d^	21.52 (6.2)	20	13–43	20.19 (6.5)	19	12–35	0.63	0.001
Multiple myeloma (n = 20)								
Inconvenience/forgetfulness	6.05 (2.8)	6	3–11	5.25 (2.3)	5	3–10	0.50	0.02
Treatment beliefs	7.95 (3.3)	8	4–19	7.25 (2.3)	8	4–10	0.25	0.29
Behavior	6.65 (2.1)	5.5	5–12	6.55 (2.3)	5	5–12	0.40	0.08
Total	20.65 (4.7)	19	15–34	19.05 (4.9)	18.5	12–30	0.58	0.007
Chronic myeloid leukemia (n = 11)								
Inconvenience/forgetfulness	6.54 (2.7)	6	3–11	6.45 (2.5)	6	3–10	0.55	0.07
Treatment beliefs	6.63 (2.5)	6	4–12	7.09 (2.8)	7	4–14	0.78	0.004
Behavior	9.90 (4.9)	9	5–21	8.63 (4.9)	6	5–17	0.27	0.43
Total	23.09 (8.3)	21	13–34	22.27 (8.4)	19	12–35	0.64	0.03

^a^ Inconvenience and forgetfulness subscale, possible range is 3 (most adherent)–15 (least adherent)

^b^ Treatment beliefs subscale, possible range is 4 (most adherent)–20 (least adherent)

^c^ Behavior subscale, possible range is 5 (most adherent)–25 (least adherent)

^d^ Total score possible ranges from 12 (most adherent)–60 (least adherent)

^e^ Pearson correlation for visit 1 and visit 2 across Likert-type range; *p *< 0.05 significance is 2-tailed

### Results

Among 31 of 42 respondents fully completing both surveys (74% response rate), most patients were receiving treatment for MM (65%), were non-Hispanic black (68%) and female (58%). Mean age at diagnosis was 59.4 years (median = 62, range 19–83 years). Mean ‘years since diagnosis’ for CML and MM patients was 3.9 (1 month to 16.2 years) and 3.0 years (< 1 month to 8.9 years), respectively (Additional file 1). The mean number of days between survey 1 and survey 2 was 136.7 days (range 25 to 287 days) and a median of 4.5 months.

Overall, 74% of patients at visit 1 and 77% of patients at visit 2 reported being adherent (total score ≤ 24 for 12 questions). In Table 1, mean and median subscale scores were approximately similar, with no mean or median subscale scores outside the adherent range. For the inconvenience and forgetfulness subscale, MM visit scores were highly correlated. For the treatment beliefs subscale, MM mean scores were lower and SDs were smaller at visit 2 compared to visit 1, indicating an improvement in treatment beliefs. For treatment beliefs, CML mean and median subscores increased between visit 1 and visit 2, indicating a decline in treatment beliefs. The CML group demonstrated improvement in medication-taking behavior. For CML patients, the behavior subscale presented the highest mean and median scores (less adherent) compared to MM patients.

In Table 2, numbers and percentages of adherent patients are presented for each question by CML and MM. In general, there was a high proportion of respondents reporting desirable scores for each of the dimensions of adherence in the ASK-12 survey at both time points. Some exceptions to these findings are mentioned in the remainder of this paragraph. The lowest adherence at visit 1 was in reply to, “I just forget to take my medicines some of the time.” All MM patients replied at both visits that cost and side effects were not factors in decision-making to skip or stop taking medication, while this finding was not repeated for CML patients. In the treatment beliefs subscale, the proportion of patients rating favorably, “My doctor/nurse and I work together to make decisions” declined for both MM and CML patients between visit 1 and visit 2.

**Table 2:** Number and percentage of adherent adult leukemia patients by ASK-12 subscale and question

Subscales	Multiple myeloma (N = 20)^a^		Chronic myeloid leukemia (N = 11)^a^	
	Visit 1	Visit 2	Visit 1	Visit 2
A. Inconvenience/forgetfulness	N (%) adherent (strongly disagree and disagree)		N (%) adherent (strongly disagree and disagree)	
Lifestyles				
1) I just forget to take my medicines some of the time	13 (65)	16 (80)	6 (55)	8 (73)
2) I run out of my medicines because I don’t get refills on time	18 (90)	18 (90)	8 (73)	9 (82)
3) Taking medicines more than once a day is inconvenient	16 (80)	16 (80)	7 (64)	8 (73)
B. Treatment beliefs	N (%) adherent (strongly agree and agree)		N (%) adherent (strongly agree and agree)	
Attitudes and beliefs				
4) I feel confident that each one of my medicines will help me	14 (70)	19 (95)^b^	10 (91)	10 (91)
5) I know if I am reaching my health goals	14 (70)	18 (90)	10 (91)	10 (91)
Help from others				
6) I have someone I can call with questions about my medicines	18 (90)	18 (90)	10 (91)	11 (100)^b^
Talking with healthcare team				
7) My doctor/nurse and I work together to make decisions	19 (95)	18 (90)	11 (100)	10 (91)^b^
C. Behavior Have You…	N (%) adherent (never or > 3 months ago)		N (%) adherent (never or > 3 months ago)	
Taking medications				
8) Taken a medicine more or less often than prescribed?	16 (80)	17 (85)	4 (36)	7 (64)
9) Skipped or stopped taking medicines because you didn’t think it was working?	19 (95)	19 (95)	9 (82)	9 (82)
10) Skipped or stopped taking medicine because it made you feel bad?	20 (100)	20 (100)	9 (82)	10 (91)
11) Skipped, stopped, not refilled or taken less medicine because of the cost?	20 (100)	20 (100)	9 (82)	9 (82)
12) Not had medicine with you when it was time to take it?	15 (75)	16 (80)	8 (73)	9 (82)

^a^ Percentages include 20 multiple myeloma or 11 chronic myeloid leukemia respondents in denominator, respectively

^b^ *p*-value < 0.05

### Discussion

The primary objective of this pilot study was to identify behaviors and barriers to oral medication adherence for adult hematology patients in two rural community-based outpatient oncology clinics. We achieved this by administering the ASK-12 survey at two time points to primarily black women with MM. Opportunities for improvement include enhancing patient–provider communication and informed decision-making. We identified that promoting strategies to remind patients to take their medications when scheduled may improve adherence. A unique contribution of this research is that the study population comprised a majority of black women with MM, which expands the limited knowledge of medication adherence in MM patients [1, 7]. Cost was not considered a barrier for adherence by MM patients.

Providers are seeking optimal approaches to improve patient adherence with oral therapies. Patients’ knowledge of their treatment regimen, consequences of non-adherence, their experience during long term treatment, and beliefs about their care team all coalesce to influence adherence [15, 20]. In a resource limited, community-based hospital where the patient population has limited literacy, we sought a short survey instrument that could be easily completed by the patient. No peer-reviewed publications were found using the ASK-12 survey to assess medication adherence in MM and CML patients [11]. A main strength of the ASK-12 survey is that subscales contain levels that address specific aspects of a barrier theme. Subscales provide summary assessment of forgetfulness and inconvenience, treatment beliefs, and behaviors, allowing providers to focus patient education in areas needing most improvement. Levels within treatment beliefs include attitudes, social support, and strong provider communication concerning treatment decisions. For example, a decline in the percentage of adherent responses to the questions, “My doctor/nurse and I work together to make decisions” suggests an educational opportunity or intervention for the healthcare team to become more engaged with patients in communicating treatment-related decision-making which may improve long-term adherence. Lower adherence in medication-taking behavior in CML patients may be a function of time since diagnosis, which was up to 16 years.

The ASK-12 survey incorporates features of the 4- or 8-question MMAS, [19, 21] and has high correlation with the 4-question MMAS on medication-taking behavior [17]. Other advantages of the ASK-12 survey include a Likert-Type response scale with darker color coding indicating non-adherence which allows immediate visualization for the provider to discuss with the patient. Gradation of darker color from least adherent to non-color as most adherent may be most similar to the Visual Analog Scale [22].

The 4 or 9-question version of MMAS is commonly implemented in clinical studies [13, 19], which may over-estimate nonadherence [21]. Some authors used a 9-item MMAS, score ranging from 1 to 13, where a score ≤ 10 indicated non-adherence and ≥ 11 indicated adherence [19]. Like the 4-item MMAS, a 9-item MMAS [21] measures overall adherence, omitting attitudes and beliefs as possible barriers to adherence [11]. Most studies which used MMAS, include only ‘yes’ or no’ responses for each question and assess adherence as binary (i.e. if patient was adherent or not) [19, 21]. We found that the ASK-12 survey [23] captures a variation of adherence behaviors and beliefs about the care team while including barriers in medication-taking behavior, all of which can assist in improving the quality of cancer care in vulnerable populations, including the elderly, rural, and low income community members which we serve. Future research aims to expand testing the ASK-12 survey, including patient and provider perceptions, as an efficient and robust approach to gain a variety of information in one sitting. The ASK-12 survey may expand future critical assessments and evaluations related to oral chemotherapy medication adherence.

## Limitations

- This project was not designed to test an educational intervention and did not include data on disease progression, molecular marker status, or assess provider perceptions of patient adherence.
- This project included a convenience sample of both newly diagnosed patients just starting therapy as well as previously diagnosed and treated patients; adherence response likely varies by duration of treatment.
- Self-reported surveys that require recalling activities days to weeks in the past may result in an over or under estimation of adherence. Median duration between surveys was 4.5 months which may impact accuracy of recalling adherence.
- Eleven patients, who did not fully complete a visit 2 survey, may have been more non-adherent, resulting in higher adherence in the remaining visit 2 respondents.
- Several follow-up surveys were obtained over the phone, which might alter patients’ responses relative to completion in-person.
- Telephone interviewing did not include a validated phone script, which could have introduced interviewer bias, resulting in patients’ reporting either higher or lower adherence.
- We were unable to compare patients who arrived for baseline visit 1 with patients who did not arrive for their appointment. Persons attending an appointment may be more adherent than those who did not.
- While we did not collect information on patient perceptions of the survey, those administering the survey felt that asking patients about barriers was an indirect way to stimulate patient’s thinking about medication adherence.

## Additional file

**Additional file 1.** Characteristics of adults with chronic myeloid leukemia (CML) and multiple myeloma (MM). The data are descriptive features of participants.

## Abbreviations

CML: chronic myeloid leukemia
MM: multiple myeloma
