# Supplementary material for: Exploring behaviors, treatment beliefs, and barriers to oral chemotherapy adherence among adult leukemia patients in a rural outpatient setting
Source: BMC Res Notes. 2018 Nov 29;11:843. doi: 10.1186/s13104-018-3935-z (PMC6267791; doi:10.1186/s13104-018-3935-z)
Supplement: Supplementary file 1 — Additional file 1. Characteristics of adults with chronic myeloid leukemia (CML) and multiple myeloma (MM). The data are descriptive features of participants. [file 13104_2018_3935_MOESM1_ESM.docx]

Additional file 1. Characteristics of adults with chronic myeloid leukemia (CML) and multiple myeloma (MM)

| **Additional file 1.** **Characteristics of adults with chronic myeloid leukemia (CML) and multiple myeloma (MM)** | | | |
| --- | --- | --- | --- |
| Characteristic |  | CML (n=11) | MM (n=20) |
| Age at Diagnosis | <40 | 3 (27) | 0 |
|  | 40-64 | 6 (55) | 10 (50) |
|  | ≥65 | 2 (18) | 10 (50) |
| Sex | Women | 8 (73) | 10 (50) |
|  | Men | 3 (27) | 10 (50) |
| Race | Black | 6 (55) | 15 (75) |
|  | White | 5 (45) | 5 (25) |
| Mean Yrs since diagnosis (SD)* |  | 3.9 (4.5) | 3.0 (2.7) |

Number (%), * mean (standard deviation)
